# Zizybeoside II From Jujube Fructus Prolongs Lifespan and Mitigates Alzheimer's Disease Progression in *Caenorhabditis elegans* Through HSF‐1/HSP‐16.2 Pathway

**DOI:** 10.1002/fsn3.72151

**Published:** 2026-07-24

**Authors:** Pu‐Sen Li, Yang Liu, Meng‐Rui Zhao, Ya‐Qiong Zhu, Ying‐Xin Shi, Meng‐Dan Jing, Xue‐Wei Hu, Chao‐Huai Jiang, Xu‐Dong Liu, Yi‐Lin You, Chang‐Jing Wu, Na‐Na Bie, Xiao‐Meng Liu

**Affiliations:** ^1^ Department of Nutrition and Food Hygiene, College of Public Health Henan Medical University Xinxiang Henan China; ^2^ Institute of Translational Medicine Zhoukou Normal University Zhoukou Henan China; ^3^ School of Life Sciences and Technology Henan Medical University Xinxiang Henan China; ^4^ College of Food Science and Nutritional Engineering, Beijing Key Laboratory of Viticulture and Enology China Agricultural University Beijing China

**Keywords:** β‐amyloid, alzheimer's disease, *Caenorhabditis elegans*, lifespan, p‐tau, zizybeoside

## Abstract

Jujube ( *Ziziphus jujuba* Mill.) has been consumed as a nutraceutical and medicinal food in China for millennia. Recent studies have highlighted the nutritional and bioactive properties of jujube fruit extracts, with potential to mitigate Alzheimer's disease (AD) progression through longevity‐enhancing effects. Our previous study explored the lifespan‐extending mechanisms of Jujube Fructus extract (JE) in *Caenorhabditis elegans* ( *C. elegans* ). Here, we evaluated the efficacy of zizybeoside II (ZB), separated from JE through HPLC, in extending lifespan and ameliorating AD pathology in *C. elegans* . The results showed that ZB treatment significantly prolonged *C. elegans* lifespan while maintaining reproductive capacity, with concurrent enhancements in normal activity and stress resistance during aging. Additionally, we found that ZB effectively alleviated various β‐Amyloid (Aβ)‐induced neurotoxic phenotypes in AD worms, including paralysis and behavioral deficits. Mechanistic investigations in N2a/APP695 cells demonstrated that ZB reduced the accumulation of Aβ and phosphorylated tau (p‐Tau) proteins, diminished oxidative stress levels through the HSF‐1/HSP16.2 pathway, and thereby slowed the progression of AD. This study provides a theoretical basis for the subsequent clinical trials of ZB in delaying AD.

## Graphical abstract

Zizybeoside II attenuates paralysis progression, boosts cognitive function, prolongs the lifespan of AD mode *C. elegans,* and reduces the accumulation of Aβ and p‐Tau proteins in AD cell models in a manner dependent on the HSF‐1/HSP16.2 pathway.

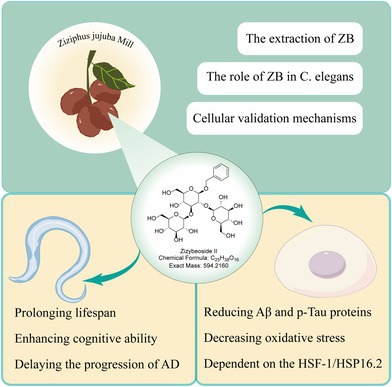

## 1 Introduction

Aging is a complex and progressive process of functional degradation that occurs at the cellular and organismal levels (Sarkar et al. 2020; Voisin et al. 2020). The biomarkers of cellular senescence are mainly as follows: epigenetic changes, genetic instability, telomere shortening, nuclear body disorders, cell cycle arrest, mitochondrial malfunction, proteostatic stress, metabolic alterations, signaling pathway rerouting, and senescence‐associated secretory phenotype (SASP) (Childs et al. 2017; López‐Otín et al. 2023; Zhang et al. 2025). The process of aging contributes to the development of age‐related diseases, such as Alzheimer's disease (AD), a neurodegenerative disorder characterized by progressive memory loss and cognitive impairment in the elderly (Scheltens et al. 2021; Stern 2012; Zhang, Cai, et al. 2024). Normally, the diagnosis of AD is defined by the presence of amyloid β (Aβ) and phosphorylated tau (p‐Tau) (Song et al. 2023). In 2018, Alzheimer's Disease International estimated a dementia prevalence of about 50 million people worldwide, projected to triple by 2050 (Wu et al. 2023). Therefore, there is an urgent need to develop new approaches for the prevention and treatment of AD.

*Ziziphus Jujuba* , also referred to as Chinese dates, belongs to the Rhamnaceae family (Hua et al. 2022). As a traditional medicinal and eidible homologous substance, Jujube is known throughout the world due to its high nutritional and health values (Gao et al. 2013; Yang et al. 2021). Today, the jujube plant is distributed widely not only in China but also in other countries (Li et al. 2021). Current studies have shown that jujube exhibits the effects of anti‐aging, anti‐oxidation, anti‐inflammation, neuroprotection, anti‐virus, and enhancing immune function (Ghimire and Kim 2017; Gupta and Gupta 2016; Park et al. 2019; Ruan et al. 2023, 2021; Yang et al. 2024; Zhu et al. 2024). Currently, there is an abundance of research on the treatment methods for AD (Cummings et al. 2023; Francesca et al. 2023; Peng et al. 2022; Se Thoe et al. 2021). However, little is known about the active substances in jujube for treating AD. In our previous study, we found that Jujube, a traditional Chinese medicinal material, could significantly prolong the lifespan of *Caenorhabditis elegans* by its anti‐aging effects (Zhang, Li, et al. 2024). To explore the effective components and efficacy of the Jujube fructus extract, we screened ZB (a small molecule substance from the crude extract of Jujube) to investigate its potential anti‐aging and anti‐AD effects.

## 2 Materials and Methods

### 2.1 *C. elegans* Strains and Maintenance Conditions

The *C. elegans* used were N2: Bristol (wildtype), which was obtained from Shanghai Tech University. The PS3551: hsf‐1 (sy441), CL2070: hsp‐16.2 (dvIs70) and CL4176: smg‐1 (cc546) were obtained from Tongji University of Life Sciences and Technology. Ethical approval was not required for this study. *C. elegans* is an invertebrate nematode and therefore exempt from the requirement for ethical review and approval by an Institutional Animal Care and Use Committee (IACUC). All the strains were maintained using standard conditions at 20°C on NGM (nematode growth medium) plates, except for CL4176, which was kept at 15°C. Worms were also allowed to grow in liquid S‐medium with concentrated *Escherichia coli* OP50 (6 mg/mL) as a food resource. Eggs were extracted from the worms before all experiments to synchronize the worms. Worms at the spawning stage were collected in M9 buffer solution (containing 0.5 M NaOH and 0.8% NaClO), digested for 3–5 min, and centrifuged to remove the supernatant at 1300× *g* for 30 s. The worms were washed twice with M9 buffer solution to retain the centrifuged precipitation. When the worms reached stage L1, live or dead (heat inactivated) *E. coli* OP50 was added to the NGM plates as food to feed the worms. OP50 was killed by heat shock temperature control 75°C during 2 h. In the L4 stage, different concentrations of ZB were added to the culture, and 40 μM Fluoro‐2′‐deoxy‐β‐uridine (FUDR, Sigma‐Aldrich) was also necessary to inhibit the growth of progeny (Stiernagle 2007).

### 2.2 Lifespan Assay

N2, CF1038, DA1116, PS3551, CB1370, EU31, and CL2070 lifespan assays were carried out at 20°C. On day 2 of adulthood, an age‐synchronized population of worms was transferred to NGM plates containing ZB (50 μg/mL, 100 μg/mL, 200 μg/mL) or an equal volume of sterile water (vehicle group). Each 3.5 cm plate was placed with 16–20 worms. Worms were transferred to new plates and scored every 2 days. Worms were scored as alive until there was no movement after repeated prodding (Wilhelm et al. 2017).

### 2.3 Oxidative Stress Resistance

On day 2 of adulthood, an age‐synchronized population of worms was transferred to S‐medium containing FUDR (40 μM) (Sigma‐Aldrich) and with 100 μg/mL ZB or vehicle treatment, respectively. After 2 days, the 5 mM paraquat was added to S‐medium, which induces lethal oxidative stress. The vitality of the worms was examined every 5 days until all worms died. Triplicate plates were used for each group. Each group included 150 worms (Wang et al. 2018).

### 2.4 Heat Stress Resistance

For the heat stress resistance assay, on day 2 of adulthood, an age‐synchronized population of worms was treated with 100 μg/mL ZB or vehicle for 2 days. The OP50 (killed by heat shock temperature control 75°C for 2 h) was added to NGM plates. Worms were placed on NGM plates containing FUDR (40 μM) in 37°C conditions, and then the number of worms was counted for 3 h until all worms died (Yang et al. 2018).

### 2.5 UV Stress Resistance

On day 2 of adulthood, an age‐synchronized population of worms was treated with 40 μM FUDR and 100 μg/mL ZB or vehicle control for 2 days for induction. Expose the worms to UV radiation for 30 min (mark this time point as Day 0). Monitor and record the number of surviving worms every 3 days until all worms died (Shi et al. 2023).

### 2.6 Movement Assay

To measure the body bending frequency and head swinging frequency, synchronized 2‐day‐old adult worms were placed on NGM plates containing FUDR (40 μM). The worms were treated with 100 μg/mL ZB or vehicle control. The body bending frequency and head swinging frequency of the nematodes per minute or 20 s were measured on days 2, 5, and 7 (Yu et al. 2024b).

### 2.7 Pharyngeal Pumping Rate

To detect the pharyngeal pumping rate, synchronized adults were randomly selected to measure the pharyngeal pumping rate every 10 s or 20 s after being treated with 100 μg/mL ZB or vehicle on days 2, 5, and 7 (Yu et al. 2024b).

### 2.8 Fertility Measurement

To determine reproductive capacity, worms were transferred to NGM plates without FUDR, with 10 individuals per group in separate plates, and treated with 100 μg/mL ZB or vehicle for 2 days. The worms were then transferred to new NGM plates every 24 h, and the number of eggs laid per day was recorded until sterility occurred (Shi et al. 2023).

### 2.9 Body Length Assay

Synchronized N2 eggs were transferred to NGM agar plates containing 100 μg/mL ZB or vehicle and cultured until the adult stage. Observation was carried out under bright‐field illumination using an Olympus BX61 fluorescence microscope (made in Japan). The body length of the worms was analyzed using ImageJ software (Yu et al. 2024b).

### 2.10 Quantitative Real‐Time PCR

The contemporaneous 2‐day‐old worms were incubated in NGM containing the same concentration of ZB for 7 days. After washing with M9 buffer, *C. elegans* were collected into 1.5 mL tubes. RNA was extracted using the TransZol Up (Transgen) and stored at the temperature of −80°C. Complementary DNA was prepared using HiScript III 1st Strand cDNA Synthesis Kit (+ gDNA wiper) (Vazyme) for real‐time polymerase chain reaction (RT‐PCR). Quantitative PCR (qPCR) was performed using ChamQ Universal SYBR qPCR Master Mix (Vazyme) (Mi et al. 2022). The mRNA expression levels of downstream genes of *daf‐16* in nematodes were monitored. β‐actin was used as the housekeeping gene for normalization, and the experimental results were expressed as 2 − (ΔΔ*Ct*) values of qPCR. The primer sequence is included in Table A1.

### 2.11 Detection of hsp16.2p::GFP Expressions

Transgenic worms CL2070 (hsp16.2p::GFP) were cultured starting from the adult stage and treated with 100 μg/mL ZB or vehicle. On the 7th day after induction, individuals were randomly selected, placed on a 3% agarose pad, and anesthetized with 2% sodium azide (Yu et al. 2024a). Imaging was performed using an Olympus BX61 fluorescence microscope (made in Japan) equipped with a GFP filter (excitation wavelength 340–380 nm, emission wavelength 435–485 nm) at 10× magnification. The fluorescence intensity was quantitatively analyzed using ImageJ software.

### 2.12 Worm Paralysis Assay

To detect the paralysis rate of worms, synchronized adult CL4176 worms were placed on NGM medium containing FUDR (40 μM) and treated with 100 μg/mL ZB or vehicle. The presence of a transparent bacterial “halos” around the head of the worm (indicating insufficient body movement to obtain food) or the failure to produce a whole‐body wave motion after nose stimulation indicated paralysis (Weng et al. 2024). Observations and recordings were made daily until all worms were paralyzed.

### 2.13 Food‐Sensing Behavior Assay

Synchronized CL4176 worms were placed on NGM medium containing 100 μg/mL ZB or vehicle for 7 days. The worms were then washed and evenly suspended in M9 buffer for later use. The bottom of a clean NGM plate was divided into four quadrants with a marker, and a circle with a radius of 0.5 cm was drawn around the origin. The upper left and lower right quadrants were marked as test quadrants, and a mixture of food and sodium azide (0.5 M) in equal proportions was added to the center of these quadrants. The upper right and lower left quadrants were marked as control quadrants, and a mixture of water and sodium azide in equal proportions was added to the center of these quadrants. An appropriate amount of worms‐containing M9 solution was placed at the origin of the plate. After 60 min, the number of worms that had completely crossed the inner circle in each quadrant was counted (Weng et al. 2024). The chemotaxis index of the worms was then calculated using the following formula:

Chemotaxis Index = (# Worms in Both Test Quadrants − # Worms in Both Control Quadrants) / (Total # of Scored Worms).

### 2.14 Cell Viability Assay (CCK‐8 Assay)

N2a/APP695 cells were kindly donated by Dr. Li Jin from Zhoukou Normal University. The cells were maintained in DMEM medium (Solabio, Beijing, China) supplemented with 10% fetal bovine serum (BI, USA), 100 units·mL‐1 penicillin and 0.1 mg·mL‐1 streptomycin at 37°C in a humidified chamber of 95% air and 5% CO_2_ atmosphere. When the cells reached the logarithmic growth phase, 100 μL (1 × 10^4^ cells/mL) were added to a 96‐well plate after trypsin digestion. After the cells had stably adhered to the plate, they were cultured for 24 h under different concentrations of ZB (0, 2.5, 5, 10, 20, 40 μM). The CCK‐8 kit (Solarbio) was used and the measurement was carried out according to the instructions of the kit. Briefly, 10 μL of CCK‐8 solution was added to each well. After incubation for 4 h, the absorbance at 450 nm was measured using a microplate reader.

### 2.15 ROS Assay

Cell was grown on a 6‐well plate for 6 h and then treated with ZB (10 μM) or vehicle for 24 h at 37°C. The measurement was carried out according to the instructions of the ROS Assay Kit with CM‐H2DCFDA (Beyotime). After loading fluorescent probes H2DCFDA onto the sample, the fluorescence intensity was detected at an excitation wavelength of 495 nm and an emission wavelength of 530 nm. The observations were recorded for 90 min at intervals of 20 min. The assay was performed in triplicate independently.

### 2.16 SOD Assay

The activity of superoxide dismutase (SOD) in cells was detected using a kit (Beyotime), and the measurement was carried out according to the instructions of the kit. Cells were grown on a 6‐well plate for 6 h and then treated with ZB (10 μM) or vehicle for 24 h at 37°C. The cells were washed twice with PBS, then scraped into cold PBS from the plate. The cells in PBS were homogenized using an ultrasonic cell disruptor. The homogenate was then centrifuged at 8000× *g* for 15 min at 4°C, and the supernatant was collected for SOD activity determination.

### 2.17 Cell RNA Interfering

N2a/APP695 cells in good growth condition were used and cultured until the cell density reached around 70%–80%. AAV vectors were constructed and packaged by Genechem Co. Ltd. (Shanghai, China). AAV was added to the culture medium at a concentration of 1 × 10^7^ TU/mL. The culture dish was gently shaken to ensure full contact between the virus and the cells. The cells were then incubated in a 37°C, 5% CO₂ incubator. After 24 h of infection, the culture medium was replaced with medium containing 10 μM ZB or vehicle. The cells were collected after 72 h of infection for subsequent detection.

### 2.18 Western Blot Analysis

Total protein samples were collected and extracted with RIPA buffer (Solarbio) from cells treated with ZB (10 μM). The protein concentration was determined using a BCA protein assay kit (thermofisher). After being separated by polyacrylamide/sodium dodecyl sulfate gel electrophoresis, the proteins were transferred to a polyvinylidene fluoride membrane. The membrane was treated with a blocking solution of 5% milk in Tris‐buffered saline with 0.05% Tween 20 (TBST), followed by incubation with the primary antibody (Table A2) diluted in TBST overnight at 4°C. After washing with TBST, the membrane was incubated with HRP‐secondary antibody for 1 h. The immunoreactive bands were visualized using a chemiluminescent substrate kit (Biosharp). Band intensities were analyzed using Image J software.

### 2.19 Statistical Analysis

Statistical analyses were carried out using GraphPad 9.0. A Kaplan–Meier lifespan analysis was carried out, and *p* values were calculated using the log‐rank test. For comparisons between two independent groups, an unpaired two‐tailed Student's *t*‐test was used. For comparisons among three or more groups, a one‐way analysis of variance (ANOVA) was conducted, followed by Tukey's post hoc test for multiple comparisons. For data involving multiple groups across distinct time points, a two‐way ANOVA followed by Tukey's post hoc test was applied. In all statistical analyses, *p* < 0.05 was accepted as statistically significant (*p < 0.05, **p < 0.01, ***p < 0.001).

## 3 Results

### 3.1 Bio‐Assay Guided Isolation of Zizybeosides

The jujube fructus (5.0 kg) was extracted by the same method as our previous literature (Zhang, Li, et al. 2024), and the jujube solution was directly loaded onto a macroporous adsorption resin (AB‐8) column and allowed to adsorb for 24 h. Then, the column was eluted with deionized water, 30%, 50%, 80%, and 95% aqueous ethanol, each for five column volumes, respectively. The fractions were concentrated under vacuum conditions to the same volume as the jujube solution loading to AB‐8 column and further subjected to *C. elegans* lifespan assay (Table 1).

**TABLE 1:** Effect of jujube fractions on the mean lifespan of *C. elegans* in wild N2.

Group	Number	Mean lifespan (days)	*p* value VS Vehicle	Maximum lifespan
Vehicle	100	12.56 ± 5.22	—	26
water	100	12.51 ± 5.48	0.7789	26
30% ethanol	100	14.43 ± 5.81	0.0071	28
50% ethanol	100	11.30 ± 5.35	0.1482	25
80% ethanol	100	13.02 ± 5.05	0.9923	24
95% ethanol	100	12.51 ± 5.24	0.9371	25

The 30% ethanol eluent (320 g) showed a similar effect as the jujube total extract in prolonging the lifespan of nematodes. It was further separated by semi‐preparative high‐performance liquid chromatography (HPLC) using a Gemini C18 column (20 mm × 250 mm, 5 μm), with 20% methanol as the mobile phase at a flow rate of 10 mL/min and detection at 210 nm. Two major components were yielded and structurally characterized as zizybeoside I (1.15 g) and zizybeoside II (1.23 g, with a purity level of 96.2%) via comprehensive spectroscopic analyses, including high‐resolution electrospray ionization mass spectrometry (HRESIMS) and nuclear magnetic resonance (NMR) spectroscopy, and the spectral data are shown in Data S1. Zizybeoside II solutions can be stored at 4°C for extended periods without loss of stability. Independent *C. elegans* lifespan assays confirmed that zizybeoside II was the main primary active ingredient in jujube for prolonging the lifespan of *C. elegans* (Table 2).

**TABLE 2:** Effect of zizybeosides on the mean lifespan of *C. elegans* in wild N_2_.

Group	Number	Mean lifespan (days)	*p* value VS Vehicle	Maximum lifespan
Vehicle	100	12.82 ± 3.74	—	23
ZizybeosideI	100	13.63 ± 4.57	0.2462	23
Zizybeoside II	100	15.88 ± 4.88	0.0002	26

### 3.2 Effect of ZB on the Lifespan and Stress Resistance of *C. elegans*

**FIGURE 1:**
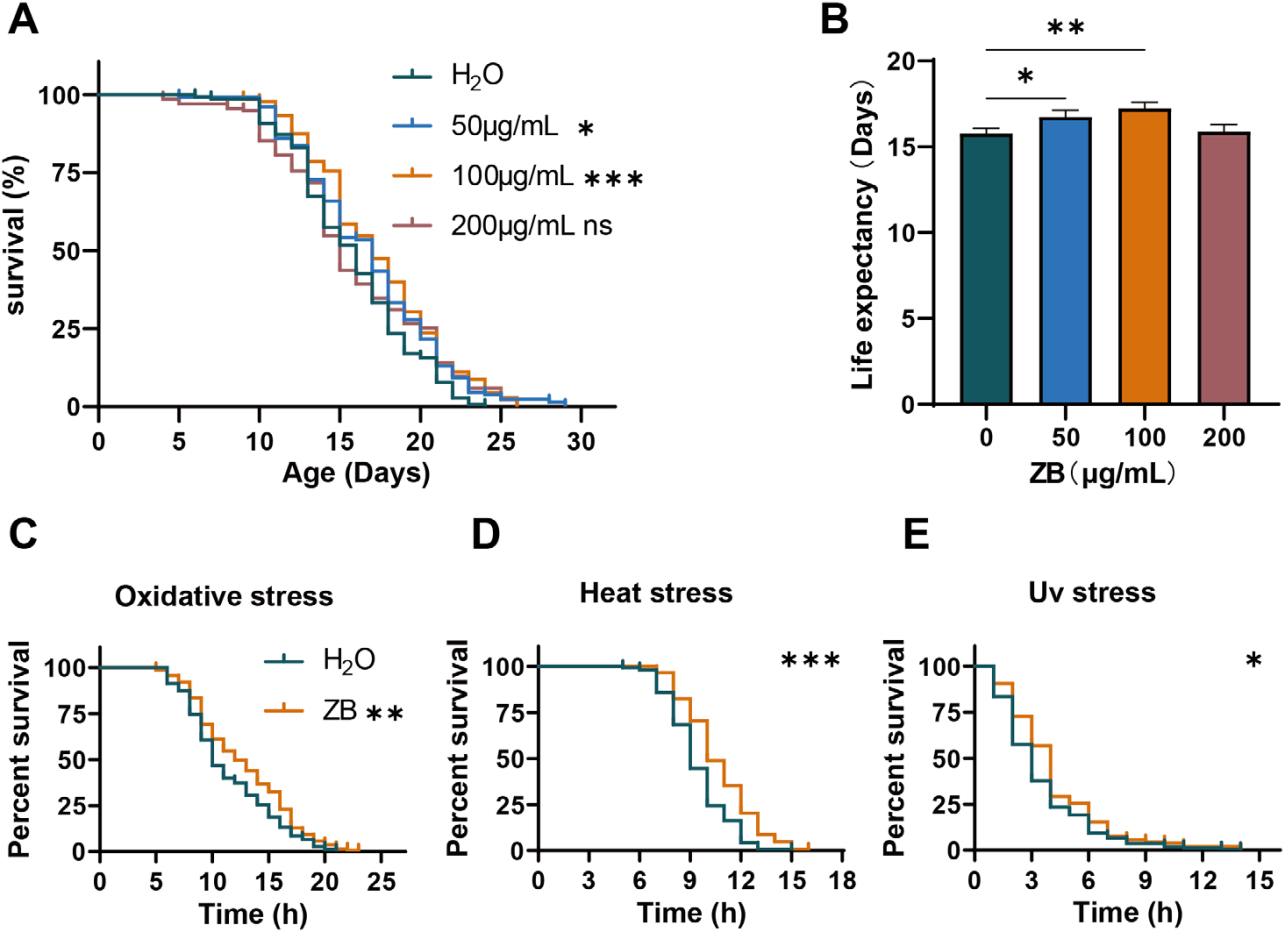
Lifespan and stress resistance of *C. elegans* exposed to ZB. (A) Lifespan of *C. elegans* treated with 50 μg/mL, 100 μg/mL, and 200 μg/mL, or H_2_O. (*n* = 150). (B) Average lifespan of *C. elegans* treated with 50 μg/mL, 100 μg/mL, and 200 μg/mL, or H_2_O. (*n* = 150). (C) Lifespan of *C. elegans* exposed to 50 μM paraquat, treated with 100 μg/mL ZB or H_2_O. (*n* = 120). (D) Lifespan of *C. elegans* exposed to 37°C thermal shock, treated with 100 μg/mL ZB or H_2_O. (*n* = 120). (E) Lifespan of *C. elegans* exposed to ultraviolet irradiation, treated with 100 μg/mL ZB or H_2_O. (*n* = 120). Statistical analysis results are shown as mean ± SE. Statistical significance was determined using a two‐tailed Student's *t*‐test. *p ≤ 0.05, **p ≤ 0.01, ***p ≤ 0.001.

To investigate the effect of ZB on the lifespan of *C. elegans* , worms were treated with various concentrations of ZB (50 μg/mL, 100 μg/mL, and 200 μg/mL), while the control group received an equivalent volume of sterile water. The concentrations of ZB were conservatively determined based on our previous in vivo finding that its source material, Jujube Fructus extract, is safe up to 1 mg/mL (1000 μg/mL) (Zhang, Li, et al. 2024). In addition, *C. elegans* has a thick, highly impermeable cuticle that severely restricts xenobiotic absorption; compounds must either penetrate this cuticle or be ingested, so the internal concentration is typically orders of magnitude lower than the external dose (Giunti et al. 2021; Wicker‐Thomas et al. 2013). This ensures all utilized concentrations fall well within the established non‐toxic window. The results demonstrated that treatment with ZB at 50 μg/mL, 100 μg/mL, and 200 μg/mL significantly prolonged the lifespan of worms compared to the control group. Specifically, the maximum lifespan increased from 24 days in the control group to 29 days, 26 days, and 26 days under the respective ZB concentrations (Figure 1A). Furthermore, the average lifespans were extended to 16.74 ± 4.38, 17.24 ± 4.11, and 15.87 ± 4.92 days, and the lifespan was increased by 6.25%, 9.43%, and 0.68%, respectively (Figure 1B). To further investigate the function of ZB, we will next treat the worms with a concentration of 100 μg/mL, which is the most effective concentration for extending worm lifespan.

Environmental stress refers to a series of nonspecific responses to physical, chemical, and biological stimuli in the internal and external environment, including oxidative stress, heat stress, and ultraviolet (UV) stress (Lin et al. 2023). Aging is often accompanied by a decline in stress resistance and adaptability (Zhou et al. 2022). In this study, *C. elegans* were exposed to oxidative stress, high temperature, and UV stress to observe the effect of ZB on the lifespan of *C. elegans* . Under oxidative stress conditions, the maximum lifespan was 21 h in the control group and increased to 23 h after ZB treatment. The mean lifespan of *C. elegans* treated with ZB was significantly increased by 11.24% compared to that of the control group (Figure 1C). Additionally, results from heat stress at 37°C and UV stress showed ZB treatment significantly enhanced the mean lifespan and maximum lifespan of *C. elegans* (Figure 1D,E).

### 3.3 Effect of ZB on the Mobility and Fertility of *C. elegans*

**FIGURE 2:**
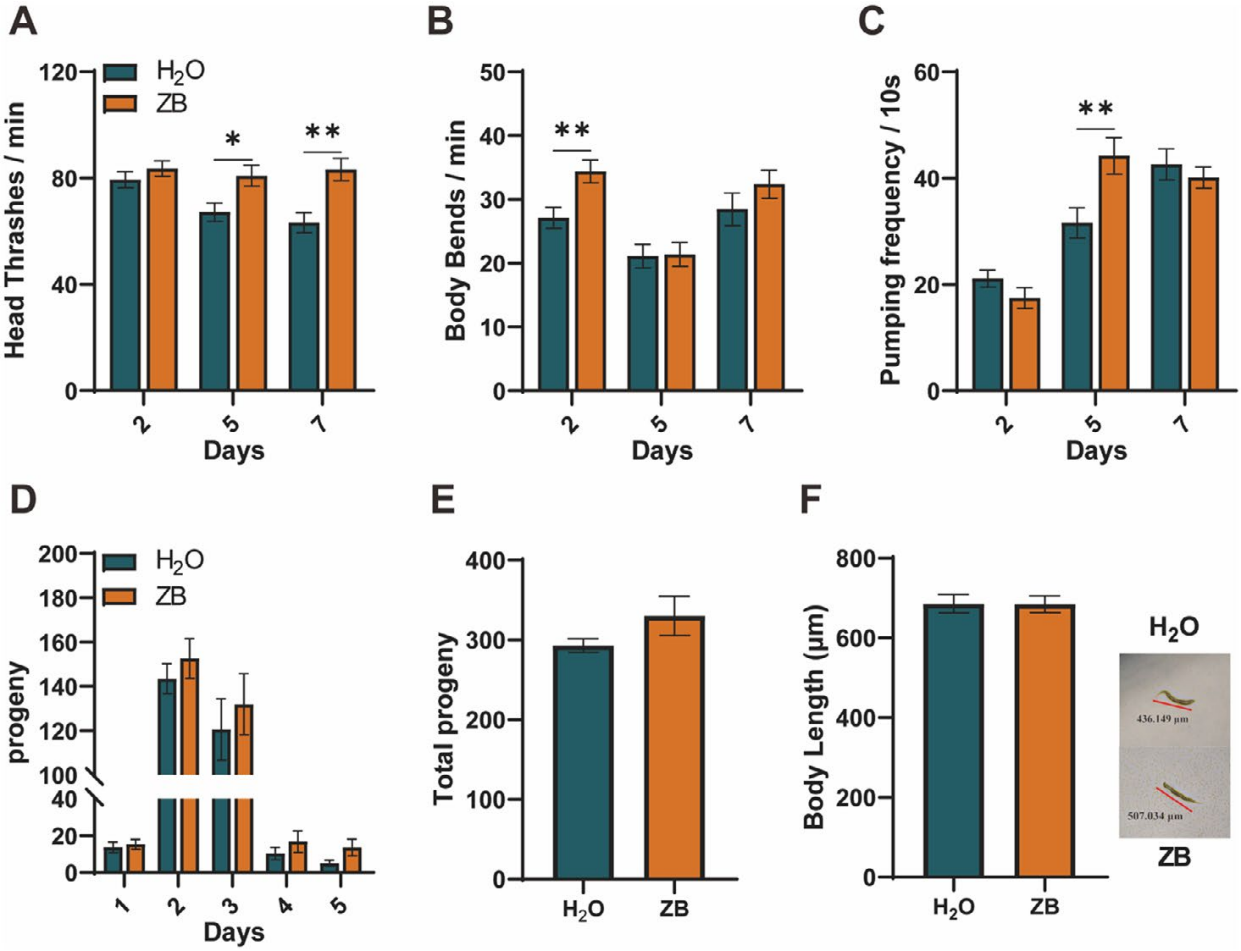
Mobility and fertility of *C. elegans* exposed to ZB. (A) The counts of head thrashes (*n* = 20). (B) The counts of body bending (*n* = 20). (C) Mean pumping rates (pumps per 10 s) are shown for each time point (*n* = 10). (D) The number of eggs laid is shown for each time point (*n* = 10). (E) The number of eggs laid of *C. elegans* treated with 100 μg/mL ZB or H_2_O (*n* = 10). (F) Body length of *C. elegans* treated with 100 μg/mL ZB or H_2_O (*n* = 10). Statistical analysis results are shown as mean ± SE. Statistical significance was determined using a two‐tailed Student's *t*‐test. *p ≤ 0.05, **p ≤ 0.01, ***p ≤ 0.001.

Aging in *C. elegans* is characterized by a decline in motility, with the presentation of key indicators of senescence including body bending, head thrashing, and swallowing behavior (Kim et al. 2017). In this study, we examined the impact of ZB treatment at 100 μg/mL on these motility parameters in *C. elegans* . The findings revealed that the ZB‐treated group exhibited a significantly higher head thrashing rate on days 5 and 7 than the control group. Specifically, following treatment with 100 μg/mL ZB, the frequency of head thrashing per minute in worms increased by 20.34% and 31.61% on days 5 and 7, respectively (Figure 2A). The results indicated a significant increase in the number of body bends per minute on day 2 after ZB treatment, though no notable changes were observed on days 5 and 7 (Figure 2B). In addition, the pharyngeal pump rate analysis showed a significant increase in the pumping frequency per 10 s on day 5 following ZB treatment compared to the control group (Figure 2C).

To determine whether ZB influences the fecundity of worms while prolonging lifespan and improving motility, we then analyzed the reproductive capacity of *C. elegans* treated with ZB at various time points. As shown in (Figure 2D–F), 100 μg/mL ZB did not significantly affect the number of eggs laid, the duration of the egg‐laying period, or the length of the worms compared to the control group. These results showed that ZB treatment could enhance motility and delay the age‐related decline of body movement in *C. elegans* , and ZB prolonged the lifespan of worms without sacrificing reproductive ability and growth.

### 3.4 ZB Requires HSF‐1/HSP‐16.2 Pathway to Extend the Lifespan of *C. elegans*

**FIGURE 3:**
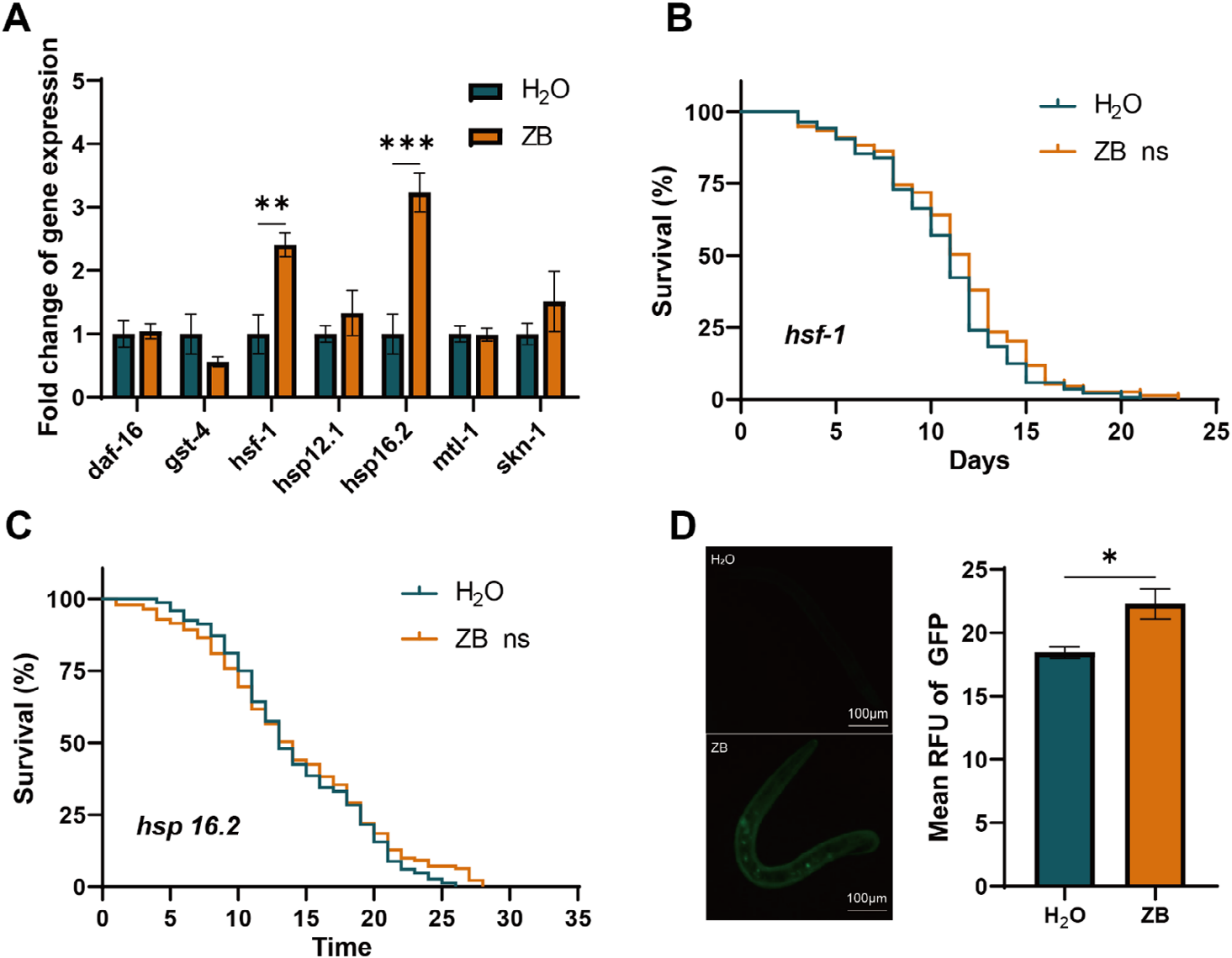
Genes affected by ZB treatment in *C. elegans* wild‐type. (A) The relative expression level of the genes related to aging or stress response in N2 strain after ZB treatment. (*n* = 6). (B) The lifespan of *hsf‐1* mutant treated with 100 μg/mL ZB or H_2_O (*n* = 129). (C) The lifespan of *hsp16.2* mutant treated with 100 μg/mL ZB or H_2_O (*n* = 129). (D) Representative images of GFP in CL2070 (*hsp16.2*‐GFP) worm treated with vehicle and 100 μg/mL ZB, the expression of HSP16.2 assay (*n* = 20). Statistical analysis results are shown as mean ± SE. Statistical significance was determined using a two‐tailed Student's *t*‐test. *p ≤ 0.05, **p ≤ 0.01, ***p ≤ 0.001.

To further elucidate the mechanism of ZB prolonging the lifespan of *C. elegans* , the RT‐qPCR was employed to evaluate their effects on the expression of genes associated with aging or stress response, including *daf‐16, gst‐4, hsf‐1, hsp12.1, hsp16.2, mtl‐1*, and *skn‐1*. The expression level of genes in untreated N2 worms was set to 1, and ZB treatment resulted in a 2.4‐fold and 3.2‐fold increase in the relative expression of *hsf‐1* and *hsp16.2*, respectively, with no effect on the expression of other genes (Figure 3A). Next, we introduced *hsf‐1* and *hsp16.2* gene‐deleted mutant worms to further explore the related mechanism. ZB treatment failed to extend the lifespan of *hsf‐1* and *hsp16.2* mutant worms (Figure 3B,C), suggesting that ZB's lifespan‐prolonging effect may partially rely on the *HSF‐1/HSP16.2* pathway. Furthermore, the GFP‐tagged *hsp16.2* transgenic strain CL2070 (HSP16.2‐GFP) was introduced to investigate whether ZB enhanced the fluorescence intensity and GFP protein levels (Figure 3D). The results demonstrated that ZB treatment significantly upregulated the transcription level of *hsp16.2*. Collectively, these findings suggest that ZB prolongs the lifespan of *C. elegans* through the *HSF‐1/HSP16.2* pathway.

### 3.5 ZB Supplementation Extends the Lifespan of AD Mode *C. elegans*

Numerous studies have demonstrated that free radicals in oxidative stress can promote the accumulation of p‐Tau and Aβ proteins, leading to neuronal degeneration, loss of function, and triggering AD (Chen and Zhong 2014). Our previous research has shown that ZB effectively mitigates the adverse effects of oxidative stress. Consequently, we explored whether ZB could alleviate AD symptoms in AD mode *C. elegans* .

**FIGURE 4:**
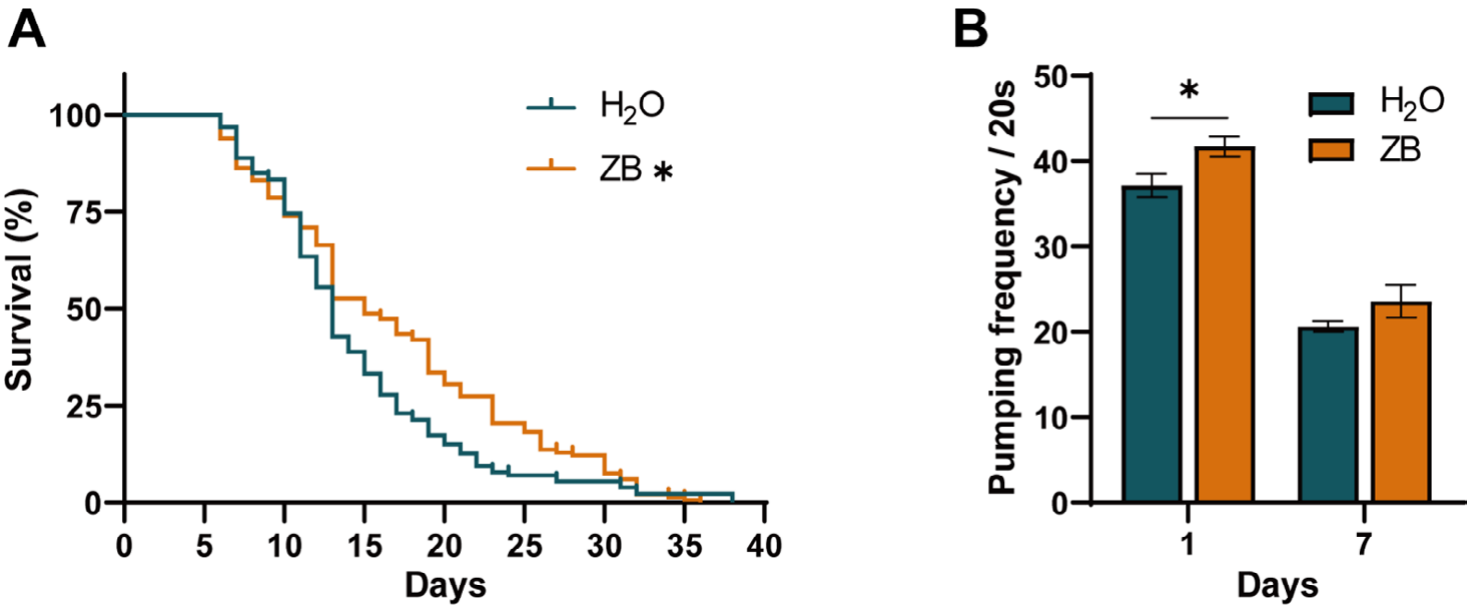
The effects of ZB on the survival and pharyngeal pumping rate of CL4176 worms. (A) The lifespan of AD *C. elegans* (CL4176) treated with 100 μg/mL ZB or H_2_O (*n* = 129). (B) Mean pumping rates (pumps per 20 s) are shown for each time point (*n* = 10). Statistical analysis results are shown as mean ± SE. Statistical significance was determined using a two‐tailed Student's *t*‐test. *p ≤ 0.05.

To investigate whether the lifespan‐extending effects of ZB could be applied to AD mode *C. elegans* , we utilized the CL4176 strain, which carries a temperature‐sensitive mutation that triggers increased Aβ peptide production at higher temperatures, subsequently leading to a paralytic phenotype (Qin et al. 2022). Consistent with our previous results with N2 mode *C. elegans* , 100 μg/mL ZB significantly extended the lifespan of AD worms, increasing their mean lifespan from 14.6 to 16.9 days (Figure 4A). A reduced pharyngeal pump rate in *C. elegans* would confer dietary restriction (DR)‐like characteristics, thereby extending the lifespan of *C. elegans* (Jiang et al. 2007). To explore this, we examined the effect of ZB on the pharyngeal pump rate in AD mode *C. elegans* . The results showed that ZB treatment significantly increased the pharyngeal pump rate on day 1, but showed no effect on day 7 (Figure 4B), suggesting that the extended lifespan of ZB treatment is not mediated via the DR pathway. We hypothesized that ZB could prolong the lifespan of AD mode *C. elegans* by ameliorating the AD‐like symptoms. Therefore, we further investigated whether ZB could exert therapeutic benefits for AD.

### 3.6 ZB Delays the Paralysis Time of AD Mode *C. elegans*

**FIGURE 5:**
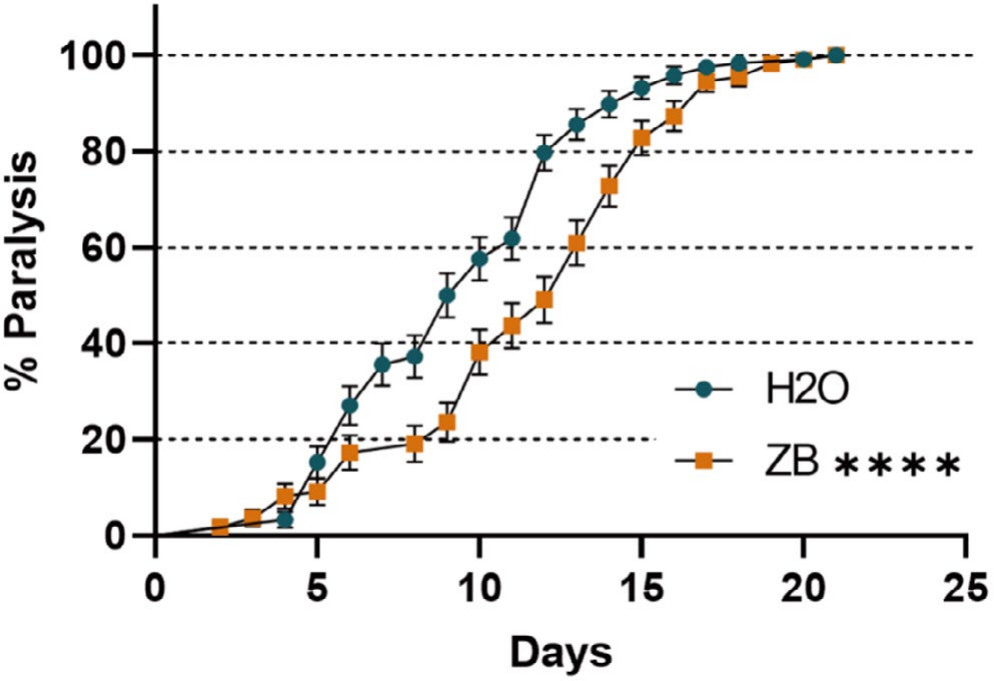
The ZB‐treated AD *C. elegans* showed delayed progression of body paralysis as compared with control worms. (*n* = 20). Statistical analysis results are shown as mean ± SE. Statistical significance was determined using a two‐tailed Student's *t*‐test. ****p ≤ 0.0001.

ZB treatment has demonstrated beneficial life‐prolonging effects in AD mode *C. elegans* , prompting us to further examine whether ZB affects paralysis induced by Aβ toxicity in the CL4176 strain. AD mode *C. elegans* were treated with either H_2_O or ZB from the early L1 stage. Subsequently, the worms were exposed to 25°C when synchronized worms reached the L4/young adult stage until all worms exhibited paralysis. The results revealed a significant delay in the onset of the paralysis phenotype in ZB‐treated AD mode *C. elegans* compared to the control (Figure 5). The results of this study demonstrate that ZB can effectively delay the progression of paralysis driven by Aβ.

### 3.7 Effects of ZB on Motor and Cognitive Abilities in AD Mode *C. elegans*

**FIGURE 6:**
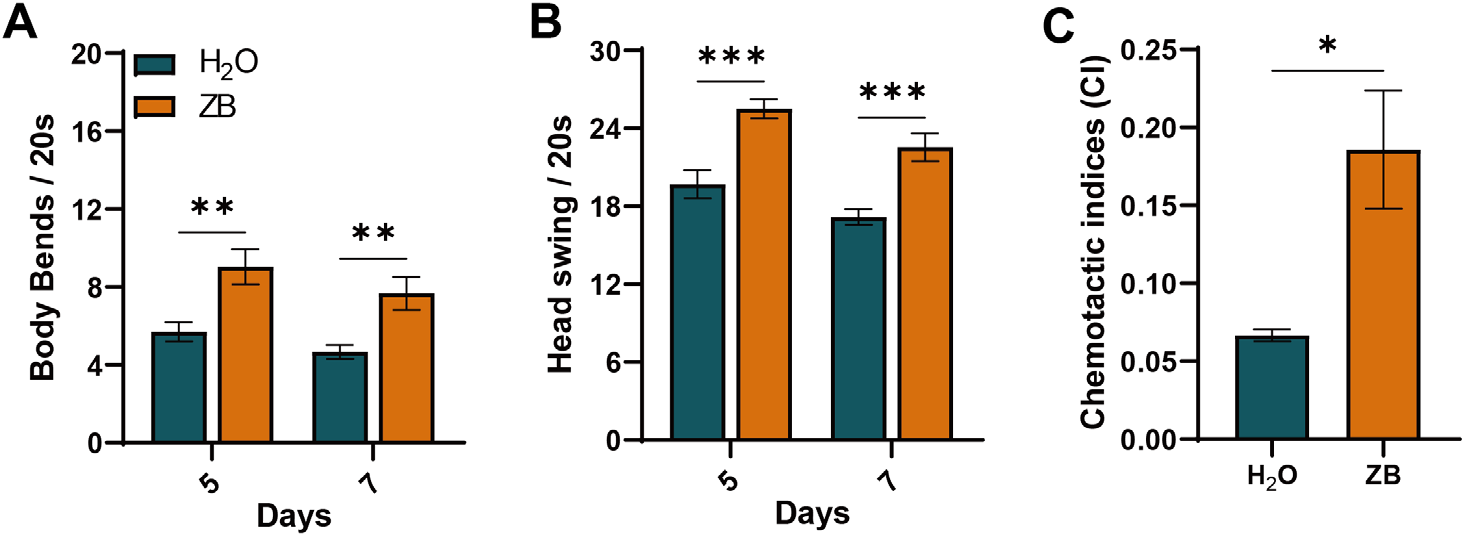
Mobility of CL4176 exposed to ZB, food chemotaxis of CL2355 exposed to ZB. (A) The counts of body bending (*n* = 30). (B) The counts of head swing (*n* = 30). (C) Food chemotic indices of CL2355 (*n* = 6). Statistical analysis results are shown as mean ± SE. Statistical significance was determined using a two‐tailed Student's *t*‐test. *p ≤ 0.05, **p ≤ 0.01, ***p ≤ 0.001.

The accumulation of Aβ protein is known to induce paralysis in AD mode *C. elegans* over time. To further investigate the impact of ZB, we evaluate its effect on the movement ability of AD mode *C. elegans* at various time points. Our results showed that worms treated with the ZB exhibited significantly elevated body bending rates on both day 5 and day 7 after treatment. Specifically, the number of body bends per 20 s increased by 1.7 and 1.4 times in worms on days 5 and 7, respectively (Figure 6A). Additionally, head swing frequency in AD mode *C. elegans* was significantly enhanced following ZB treatment compared to the control group (Figure 6B). As olfaction represents a critical sensory modality underpinning cognitive behaviors such as learning, memory, discrimination, and other neurological functions in *C. elegans* , we utilized the transgenic CL4176 strain‐engineered as an AD model expressing the human Aβ1‐42 peptide with aggregates in neuronal cells and exhibiting defective chemotaxis to assess neuron‐controlled behaviors (Guo et al. 2015). A food‐sensing behavior assay was performed to assess the effects of Aβ on neuronal function in *C. elegans* . The results demonstrated that ZB significantly enhanced the ability of *C. elegans* to explore food sources (Figure 6C). Collectively, these findings demonstrate that ZB not only improves motor function and alleviates the paralysis phenotype associated with AD in *C. elegans* , but also mitigates neurocognitive impairments induced by Aβ toxicity.

### 3.8 ZB Increased the Expression of HSF‐1 and HSP‐16.2 Genes in AD Worms

**FIGURE 7:**
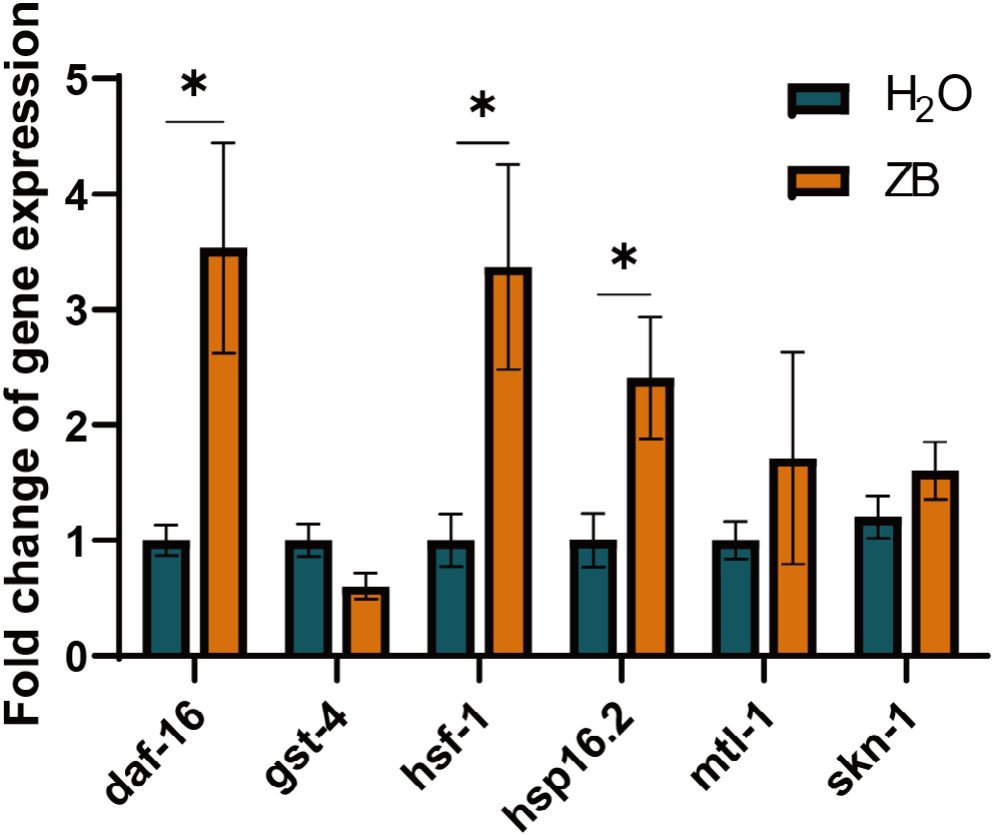
The relative expression level of the downstream gene of daf‐16 in CL4176 strain after ZB treatment. (*n* = 7). The expression levels of *daf‐16*, *hsf‐1*, and *hsp16.2* genes in the CL4176 mutant were significantly increased after ZB treatment. Statistical analysis results are shown as mean ± SE. Statistical significance was determined using a two‐tailed Student's *t*‐test. *p ≤ 0.05.

Previous studies have demonstrated that *skn‐1*, *hsf‐1*, and *daf‐16* play crucial roles in regulating Aβ aggregation (Qin et al. 2022). To investigate the molecular mechanism underlying ZB's protective effects on AD, we performed RT‐qPCR in CL4176 worms to detect *daf‐16*, *gst‐4*, *hsf‐1*, *hsp16.2*, *mtl‐1*, and *skn‐1* levels. The results showed that ZB treatment did not activate *gst‐4*, *mtl‐1*, and *skn‐1*, while it significantly increased the expression levels of the *hsf‐1*, *daf‐16*, and *hsp16.2* genes. Specifically, ZB treatment increased *hsf‐1* gene expression by 3.37‐fold, *daf‐16* gene expression by 3.53‐fold, and *hsp16.2* gene expression by 2.41‐fold compared to the control group (Figure 7). In conclusion, these findings suggest that the anti‐AD mechanism of ZB may be dependent on the *HSF‐1/HSP16.2* pathway.

### 3.9 Effects of ZB on the Survival of N2a/APP695 Cells

**FIGURE 8:**
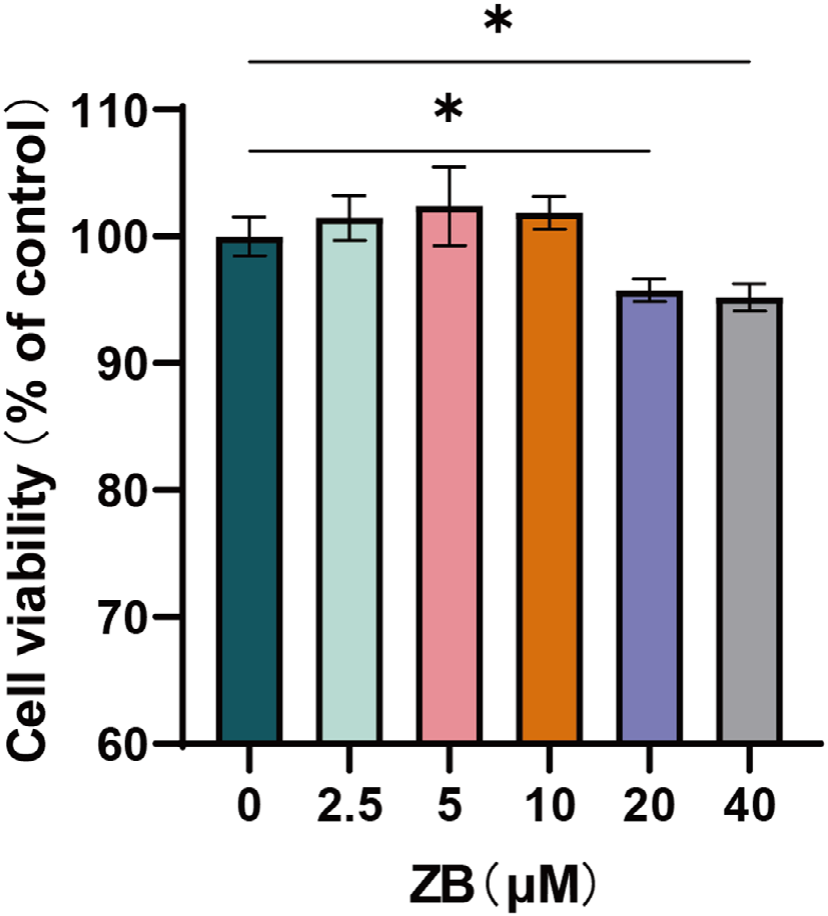
Cell viability of different concentrations of ZB‐treated N2a/APP695 cells. (*n* = 6). Statistical analysis results are shown as mean ± SE. Statistical significance was determined using a two‐tailed Student's *t*‐test. *p ≤ 0.05.

To further verify the mechanism by which ZB ameliorates AD‐like symptoms, we utilized N2a/APP695 cells and employed the CCK8 assay to evaluate the effect of various concentrations of ZB on cell survival. N2a/APP695 cells were treated with ZB at concentrations ranging from 0 to 40 μM for 24 h. The 40 μM concentration used in cell assays is equivalent to 24 μg/mL, which is lower than the dose used in *C. elegans* , because cells are directly exposed to the compound in the medium without any tissue barrier, leading to high cellular uptake. The results showed that ZB concentrations between 2.5 and10 μM had no significant toxic effect on N2a/APP695 cells, whereas concentrations of 20–40 μM reduced cell viability significantly (Figure 8). Integrating findings from our preliminary cell proliferation assays, which demonstrated that ZB at 2.5–10 μM did not induce notable damage to renal cells, we selected 10 μM ZB as the optimal concentration for subsequent experiments.

### 3.10 ZB Reduced Oxidative Response and Aβ and P‐Tau Protein Expression in N2a/APP695 Cells

**FIGURE 9:**
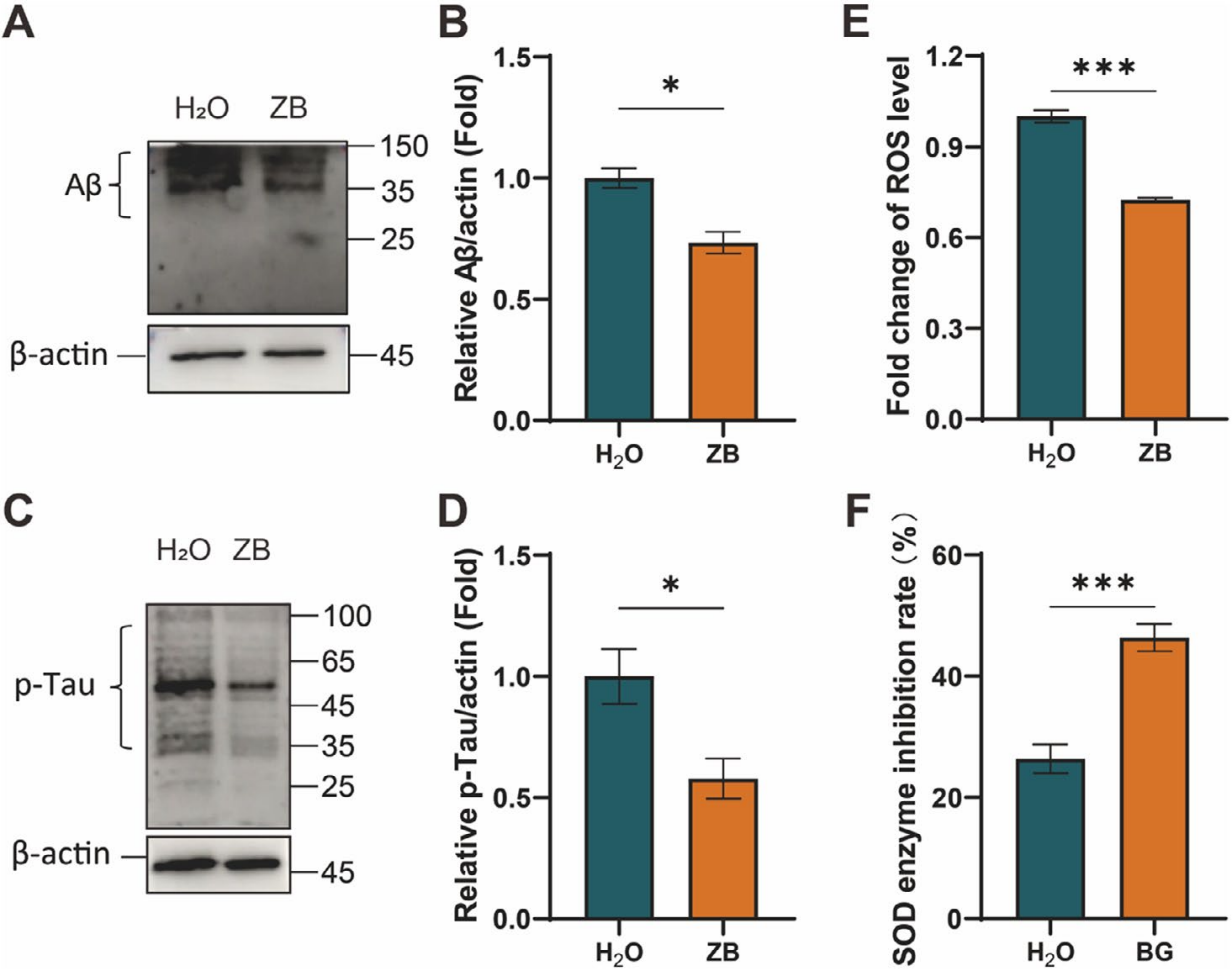
*ZB reduced oxidative resp*onse and Aβ and p‐Tau protein expression in N2a/APP695 cells. (A) Western blot was used to detect Aβ protein, and β‐Actin protein was used as an internal reference. (B) The bar graph indicates the quantification of the relative protein level of Aβ in N2a/APP695 cells (*n* = 6). Data are presented as mean ± SE. (C) Western blot was used to detect p‐Tau protein, and β‐Actin protein was used as an internal reference. (D) The bar graph indicates the quantification of the relative protein level of p‐Tau in N2a/APP695 cells (*n* = 6). Statistical analysis results are shown as mean ± SE. (E) The ROS levels in worms treated with ZB or vehicle (*n* = 6). (F) The SOD levels in worms treated with ZB or vehicle (*n* = 6). *p ≤ 0.05, ***p ≤ 0.001.

The accumulation of Aβ and p‐Tau is widely recognized as a hallmark of AD (Z. Jiang et al. 2024). To evaluate ZB's inhibitory impact on Aβ protein aggregation, we quantified Aβ oligomer levels in ZB‐treated cells using immunoblot analysis. The results demonstrated that ZB significantly reduced the abundance of Aβ oligomers compared to the control group (Figure 9A,B). Additionally, we investigated whether ZB could attenuate p‐Tau aggregation, revealing a significant reduction in p‐Tau accumulation in cells after ZB treatment (Figure 9C,D); Data S2. In conclusion, ZB treatment reduced Aβ and p‐Tau accumulation in N2a/APP695 cells. To further evaluate the anti‐oxidative effect of ZB, the intracellular reactive oxygen species (ROS) and SOD activity were measured in ZB‐treated cells. Compared to the control group, ZB markedly decreased the ROS levels while enhancing the activity of SOD, a key antioxidant enzyme (Figure 9E,F).

### 3.11 ZB Reduces Aβ Protein and Tau Accumulation Through HSF‐1/HSP16.2 Pathway

**FIGURE 10:**
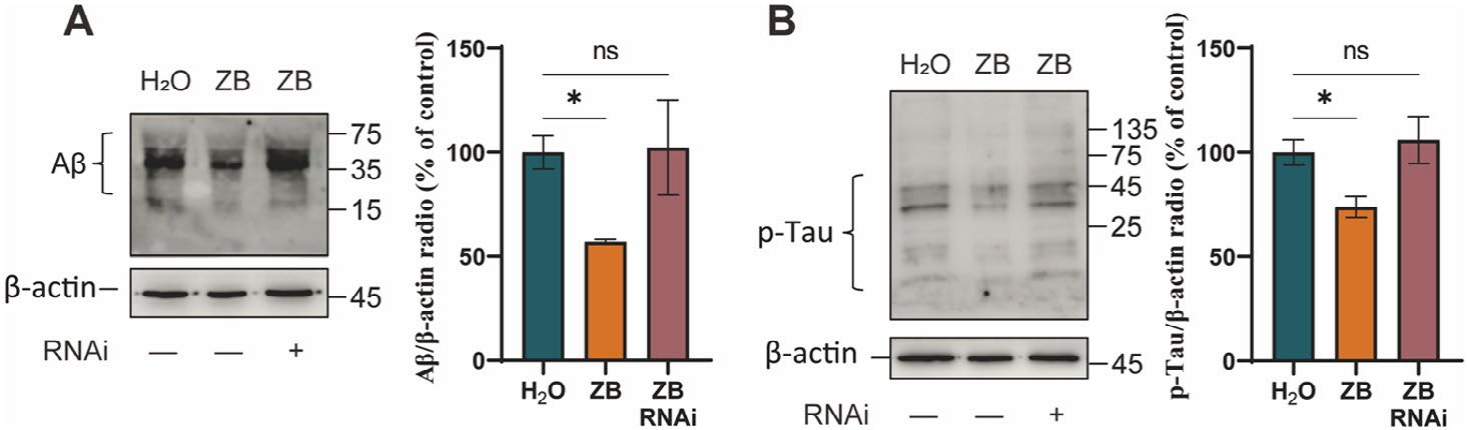
ZB reduced Aβ and p‐Tau protein expression in *hsf‐1* RNAi or *hsp16.2* RNAi N2a/APP695 cells *n* = 6. Western blot was used to detect Aβ protein, and β‐Actin protein was used as an internal reference. The bar graph indicates the quantification of the relative protein level of Aβ in different N2a/APP695 cells. Data are presented as mean ± SE (A). Western blot was used to detect p‐Tau protein, and β‐Actin protein was used as an internal reference. The bar graph indicates the quantification of the relative protein level of p‐Tau in different N2a/APP695 cells. Data are represented as mean ± SE (B). *p ≤ 0.05.

To verify the importance of *hsf‐1* and *hsp16.2* in ZB treatment of AD, we knocked down *hsf‐1* or *hsp16.2* gene expression by RNA interference (RNAi) in N2a/APP695 cells. We subsequently investigated whether *hsf‐1* RNAi or *hsp16.2* RNAi would counteract the effect of ZB in reducing Aβ and p‐Tau levels. The results indicated that, following the knockdown of *hsf‐1* or *hsp16.2*, the Aβ protein and p‐Tau protein levels did not decrease in ZB‐treated cells compared to the control group, underscoring the essential role of both *hsf‐1* and *hsp16.2* in mediating the protective effect of ZB on AD model cells (Figure 10A,B). Data S2. These observations suggest that the beneficial effects of ZB in mitigating Aβ and p‐Tau accumulation are mediated, at least partially, through the HSF‐1/HSP16.2 insulin‐like signaling pathway.

## 4 Discussion

This study centered on zizybeoside II (ZB), isolated through HPLC, which notably prolonged the lifespan of *C. elegans* while maintaining reproductive capacity and improving activity and stress resistance. ZB also decreased the levels of Aβ and p‐Tau in AD worms, thereby reducing paralysis, behavioral dysfunction, neurotoxicity, and ROS accumulation. Mechanistic analyses indicated that ZB's effects were mediated through the HSF‐1/HSP16.2 pathway, effectively attenuating AD progression.

Jujube, a common traditional Chinese medicine herb renowned for its anticancer and antiviral properties, has a long‐standing clinical history in treating various ailments (Ji et al. 2017). However, its implications for aging and AD‐related pathologies have been scarcely explored. In our previous investigation, we demonstrated that the aqueous extract of jujube fruit (JE) extended both lifespan and health span in *C. elegans* , elucidating its underlying longevity‐promoting mechanisms (Zhang, Li, et al. 2024). In the present study, two bioactive components were isolated and purified from the JE and identified as ZB I and ZB II, respectively. ZB II was identified as the principal active constituent by lifespan assessment assays, warranting further exploration of its anti‐aging effects. Our results showed that ZB II at concentrations of 50 μg/mL and 100 μg/mL significantly prolonged the lifespan of *C. elegans* , accompanied by enhanced motility and improved stress resistance under adverse conditions. Notably, mechanistic investigations demonstrated that ZB‐mediated lifespan extension in *C. elegans* is associated with the inhibition of protein aggregation via regulation of the HSF‐1/HSP16.2 signaling pathway.

Aging is one of the unmodified factors correlating with the onset of AD (Stefaniak et al. 2022). With aging, the abnormal deposition of Aβ and p‐Tau proteins in the brain is strongly linked to the pathogenesis of AD (Ciesla et al. 2024; Monllor et al. 2021; Tarawneh and Holtzman 2010). The pathogenesis of AD is complex and has long perplexed scientists. Although AD has many complex pathogenic mechanisms, oxidative stress and its damage to neurons are the core factors of all mechanisms (Sandberg et al. 2022). Our previous results showed that ZB prolongs lifespan and is related to anti‐oxidative stress, so we speculated that ZB may have a potential role in the treatment of AD in *C. elegans* . As expected, ZB could significantly prolong the lifespan of AD *C. elegans* , delay the paralysis phenotype of AD *C. elegans* , improve cognitive performance, and increase the antioxidant capacity of AD *C. elegans* .

Notably, we examined the expression levels of longevity‐related genes (*daf‐16, gst‐4, hsf‐1, hsp16.2, mtl‐1, skn‐1*) in AD worms after ZB treatment, and the results showed that ZB treatment significantly increased the expression of *hsf‐1*, *hsp16.2*, and *daf‐16*. Consistent with these findings, our in vitro studies in AD cell models demonstrated that silencing *hsf‐1* and *hsp‐16.2* via RNAi abolished the ZB‐mediated reduction of Aβ and p‐Tau proteins, underscoring the critical role of the HSF‐1/HSP‐16.2 axis. While AD pathogenesis involves a vast and complex network of inflammatory, apoptotic, and oxidative stress pathways. (Abubakar et al. 2022; Kumari et al. 2023; Sharma et al. 2019; Zhang, Zhang, et al. 2024), longevity and proteostasis pathways such as DAF‐16/FOXO and SKN‐1/Nrf2 are particularly crucial and often highly interconnected. The observed induction of *daf‐16* alongside *hsf‐1* introduces ambiguity regarding pathway specificity. However, despite the upregulation of *daf‐16*, we did not observe a significant increase in the expression of its downstream target genes following ZB treatment. This discrepancy suggests potential time‐dependent gene regulation, where varying durations of ZB intervention may lead to differential, temporal activation of these interconnected networks. Furthermore, there is likely significant crosstalk between the HSF‐1 and major insulin‐like signaling pathways (e.g., DAF‐16/FOXO). While our data strongly points to the HSF‐1/HSP‐16.2 axis as a primary mediator, we acknowledge the limitation in assigning absolute pathway specificity; ZB may exert its protective effects through a coordinated modulation of multiple longevity pathways rather than a single, isolated target. To better understand this complexity, we will further explore whether genes within the insulin‐like signaling pathway exhibit time‐specific expression profiles following ZB intervention.

In addition, to further validate the functional activation of the HSF‐1/HSP16.2 axis, we conducted Western blot analysis. While our qPCR results showed significant upregulation of hsf‐1 and hsp16.2 mRNA, total protein levels in whole‐worm lysates did not exhibit a corresponding increase (Data S3). This observation is consistent with the established regulatory model of HSF‐1. Specifically, its stress‐responsive activation is primarily mediated by post‐translational modifications and the translocation of cytoplasmic monomers into nuclear‐localized trimers, rather than an immediate increase in total cellular protein abundance (Anckar and Sistonen 2011). Since our WB assays were performed on total lysates, they reflect the stable total protein pool but do not capture these critical shifts in subcellular localization. Nevertheless, the robust induction of fluorescence in the hsp16.2::GFP reporter strain and the finding that hsf‐1 RNAi abolished ZB's neuroprotective effects collectively provide strong evidence for the functional activation of this signaling axis at the translational and physiological levels.

ZB is isolated and purified from the concentrated extract of dried red dates, a process involving drying and condensing fresh red dates. Therefore, low yield makes it difficult to produce high‐purity ZB samples on a large scale, with only milligram‐scale products typically obtainable from a single experiment. This limitation directly hinders the large‐scale implementation of pharmacological activity research, making it impossible to conduct large‐sample‐size in vivo animal experiments (such as long‐term toxicity tests and pharmacodynamic verification). Instead, researchers have to rely solely on small‐sample experiments to draw the conclusions presented in the research papers. To enhance the practical application of ZB, optimization of the production process is essential.

## 5 Conclusion

In summary, our results indicate that ZB significantly extends the lifespan of *C. elegans* and enhances its stress resistance and antioxidant capacity, markedly improving the worms' motor ability. Furthermore, AD mode *C. elegans* and AD cell models were used, and our findings suggest that ZB attenuates paralysis progression, boosts cognitive function, and reduces the accumulation of Aβ and p‐Tau proteins in a manner dependent on the HSF‐1/HSP16.2 pathway. These findings provide solid evidence for the role of ZB, a component of jujube crude extract, in promoting longevity and improving AD‐related cognitive impairments.

## Author Contributions

**Ya‐Qiong Zhu:** investigation. **Pu‐Sen Li:** investigation, writing – original draft. **Ying‐Xin Shi:** formal analysis. **Chao‐Huai Jiang:** validation. **Xu‐Dong Liu:** visualization. **Chang‐Jing Wu:** conceptualization, writing – original draft. **Xue‐Wei Hu:** validation. **Yi‐Lin You:** resources. **Meng‐Dan Jing:** formal analysis. **Xiao‐Meng Liu:** conceptualization, writing – original draft, funding acquisition. **Yang Liu:** visualization. **Meng‐Rui Zhao:** investigation. **Na‐Na Bie:** writing – original draft, writing – review and editing.

## Funding

This work was supported by the Starting Research Fund from the XinXiang Medical University (XYBSKYZZ202135).

## Conflicts of Interest

The authors declare no conflicts of interest.

## Supporting information


**Data S1:** Supporting Information.
**Data S2:** Supporting information.
**Data S3:** Supporting information.
**Figure S1:** HPLC analyses of 30% ethanol elution of jujube extraction (a, 20% methanol as mobile phase on a C18 column detected at 210 nm) and yielded zizybeoside II with a purity level of 96.2% (b, 10% methanol as mobile phase).

## Data Availability

The data that support the findings of this study are available from the corresponding author upon reasonable request.

## Appendix A Appendices

**TABLE A1:** List of primers used for qRT‐PCR assays.

Gene's name	Forward (5′‐3′)	Reverse (5′‐3′)
*β‐Actin*	GCTCTTGCCCCATCAACCAT	GCCGGACTCGTCGTATTCTT
*daf‐16*	TCAGATGGTAGCGGCGAATC	TACATTGCTCGAAGTGCCGA
*hsp16.2*	TCCATCTGAGTCTTCTGAGATTGTT	TGAGACGTTGAGATTGATGGCA
*gst‐4*	AAGCTGAAGCCAACGACTCC	AATGGGAAGCTGGCCAAATG
*hsf‐1*	TTGACGACGACAAGCTTCCAGT	AAAGCTTGCACCAGAATCATCCC
*hsp12.1*	ATACATATGCACACAATTCCAATTACAACTG	TACTCGAGAAGTTTATTGGCAGTGATTG
*mtl‐1*	GGCTTGCAAGTGTGACTGC	TCTCCGCACTTGCATTGCTT
*skn‐1*	AACGAAGACGAAGACAGTGCT	TCCAGATGAATATGGACGACAC

**TABLE A2:** List of antibodies for Western blot.

Name	Dilution	Source	Identifier
Beta Actin Antibody	1:5000	Affinity	AF7018
Phospho‐TAU (Ser404) Recombinant antibody	1:2000	Proteintech	81,383–1‐RR
Purified anti‐β‐Amyloid, 1–16 Antibody	1:1000	BioLegend	SIG‐39320
