# Supplementary material for: Zizybeoside II From Jujube Fructus Prolongs Lifespan and Mitigates Alzheimer's Disease Progression in Caenorhabditis elegans Through HSF‐1/HSP‐16.2 Pathway
Source: Food Sci Nutr. 2026 Jul 24;14(7):e72151. doi: 10.1002/fsn3.72151 (PMC13398156; doi:10.1002/fsn3.72151)
Supplement: Supplementary file 1 — Data S1: Supporting Information. Data S2: Supporting information. Data S3: Supporting information. Figure S1: HPLC analyses of 30% ethanol elution of jujube extraction (a, 20% methanol as mobile phase on a C18 column detected at 210 nm) and yielded zizybeoside II with a purity level of 96.2% (b, 10% methanol as mobile phase). [file FSN3-14-e72151-s001.docx]

**Supplementary_Material-S1**

**Spectral data of compounds**

Zizybeoside I: white amorphous powder, molecular formula C_19_H_28_O_11._ Positive HR-ESI-MS: *m/z* measured 450.2003 [M + NH_4_]^+^, calcd for C_19_H_28_O_11_NH_4_ [M + NH_4_]^+^ 450.1975; measured 455.1528 [M + Na]^+^, calcd for C_19_H_28_O_11_Na [M + Na]^+^ 455.1529. ^1^H-NMR (500 MHz, DMSO-*d*_6_) δ: 7.43 (d, *J* = 7.5 Hz, 2H), 7.32 (t, *J* = 7.4 Hz, 2H), 7.25 (t, *J* = 7.2 Hz, 1H), 5.57 (d, *J* = 3.3 Hz, 1H), 5.34 (d, *J* = 3.5 Hz, 1H), 5.08 (d, *J* = 5.0 Hz, 1H), 4.95 (d, *J* = 3.9 Hz, 1H), 4.88 (d, *J* = 4.5 Hz, 1H), 4.85 (d, *J* = 12.1 Hz, 1H), 4.61 (d, *J* = 12.2 Hz, 2H), 4.57 (t, *J* = 5.9 Hz, 1H), 4.45 (t, *J* = 8.2 Hz, 3H), 4.37 (t, *J* = 5.6 Hz, 1H), 3.73-3.67 (m, 2H), 3.64-3.58 (m, 1H), 3.48 (dt, *J* = 12.1, 6.2 Hz, 3H), 3.40 (dd, *J* = 8.3, 3.9 Hz, 1H), 3.36 (d, *J* = 7.5 Hz, 1H), 3.16 (p, *J* = 2.5 Hz, 5H), 3.11-2.99 (m, 3H). ^13^C-NMR (125Hz, DMSO-*d*_6_) δ: 60.74, 60.94, 69.57, 69.65, 69.79, 75.01, 76.12, 76.20, 76.80, 77.05, 82.33, 100.71, 104.37, 127.15, 127.17 (2C), 128.06 (2C), 138.12.

Zizybeoside II: white amorphous powder, molecular formula C_25_H_38_O_16._ Positive HR-ESI-MS: *m/z* measured 612.2531 [M + NH_4_]^+^, calcd for C_25_H_38_O_16_NH_4_ [M + NH_4_]^+^ 612.2504; measured 617.2086 [M + Na]^+^, calcd for C_25_H_38_O_16_Na [M + Na]^+^ 617.2058. ^1^H-NMR (500Hz, DMSO-*d*_6_), δ:7.41 (d, *J* = 7.1 Hz, 2H), 7.32 (t, *J* = 7.4 Hz, 2H), 7.26 (t, *J* = 7.3 Hz, 1H), 5.58 (d, *J* = 4.9 Hz, 1H), 5.09 (d, *J* = 5.8 Hz, 2H), 5.03 (d, *J* = 5.5 Hz, 1H), 4.91-4.85 (m, 3H), 4.66-4.60 (m, 5H), 4.50 (d, *J* = 6.9 Hz, 1H), 4.42 (d, *J* = 7.9 Hz, 1H), 4.33 (t, *J* = 5.6 Hz, 1H), 3.70 (dd, *J* = 11.5, 5.2 Hz, 2H), 3.58 (q, *J* = 7.8, 6.6 Hz, 3H), 3.52-3.48 (m, 1H), 3.40 (dt, *J* = 11.7, 5.9 Hz, 2H), 3.26-3.22 (m, 2H), 3.19 (dtd, *J* = 13.3, 8.7, 4.8 Hz, 1H), 3.15-3.04 (m, 3H), 3.01 (ddd, *J* = 7.1, 5.5, 3.4 Hz, 1H), 2.95 (td, *J* = 8.4, 6.0 Hz, 1H). ^13^C-NMR (125Hz, DMSO-*d*_6_) δ: 60.86, 61.00 (2C), 68.48, 69.53, 70.07 (2C), 73.71, 74.53, 76.32 (2C), 76.49, 76.80, 76.96, 79.08, 86.39, 100.97, 102.73, 103.31, 127.18, 127.20 (2C), 128.05 (2C), 138.18.

**HPLC analyses of compounds**


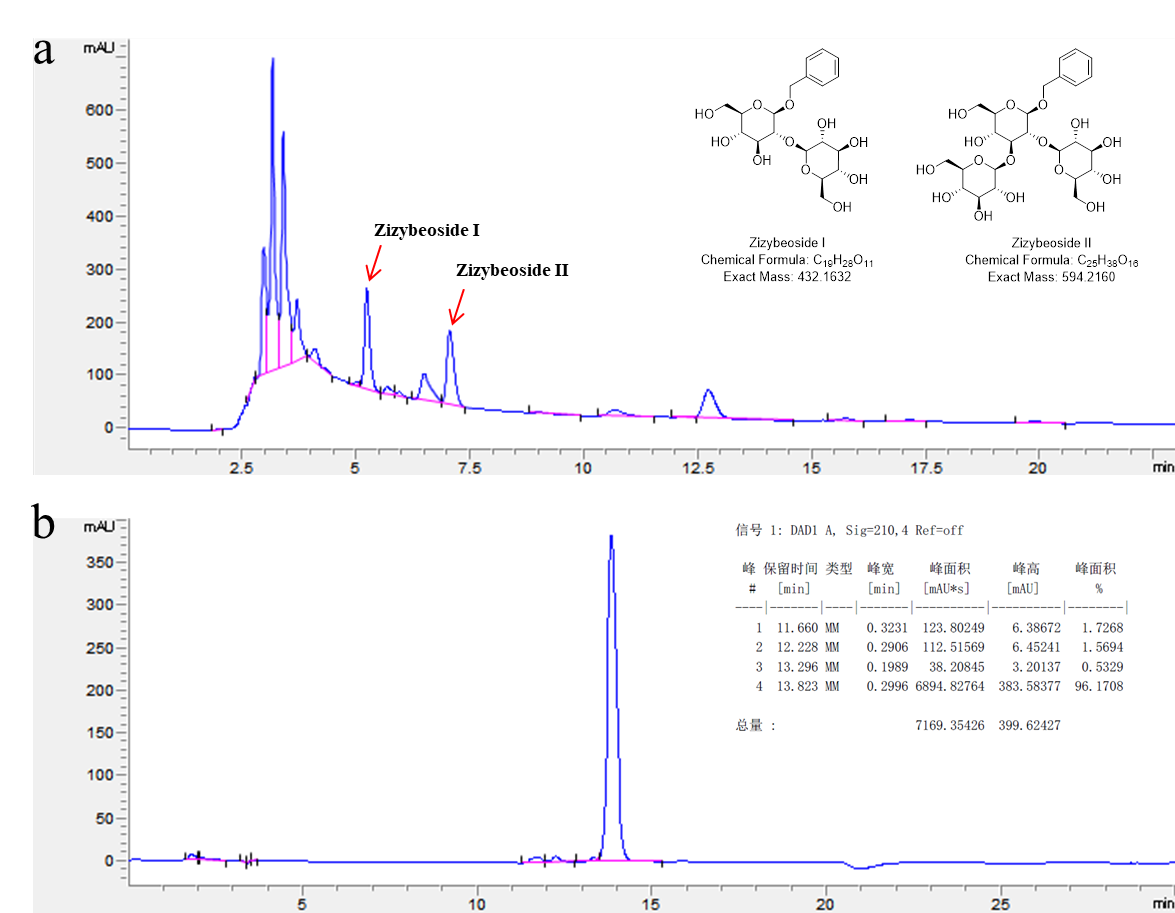


Figure S1. HPLC analyses of 30% ethanol elution of jujube extraction (a, 20% methanol as mobile phase on a C18 column detected at 210 nm) and yielded zizybeoside II with a purity level of 96.2% (b, 10% methanol as mobile phase).

**MS and NMR** **Spectra of compounds**


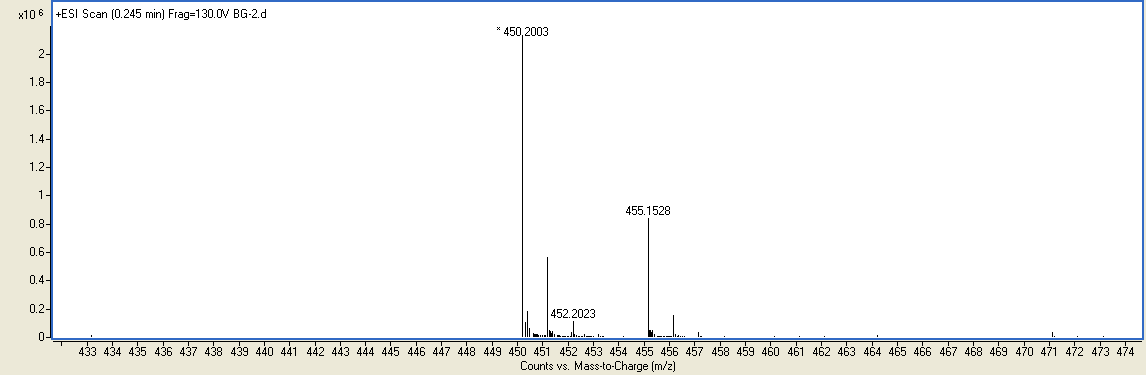


HR-ESI-MS spectrum of Zizybeoside I

^1^H-NMR Spectrum of Zizybeoside I in DMSO-*d*_6_ (500 MHz)

^13^C-NMR Spectrum of Zizybeoside I in DMSO-*d*_6_ (125 MHz)


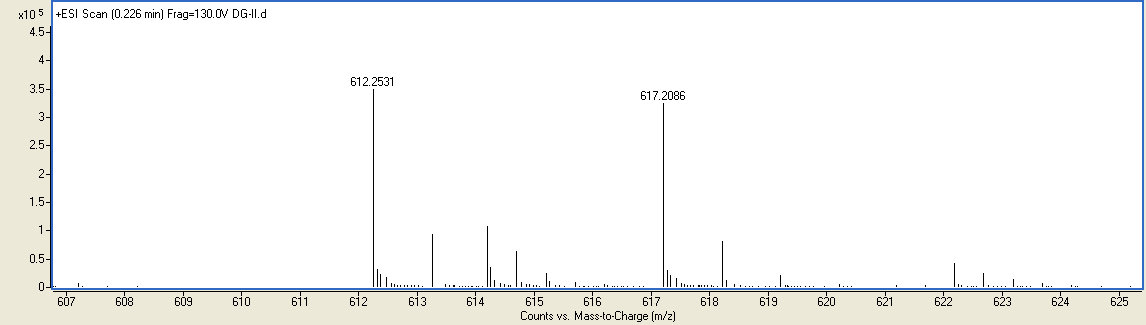


HR-ESI-MS spectrum of Zizybeoside II

^1^H-NMR Spectrum of Zizybeoside II in DMSO-*d*_6_ (500 MHz)

^13^C-NMR Spectrum of Zizybeoside II in DMSO-*d*_6_ (125 MHz)

**Supplementary_Material-S2**


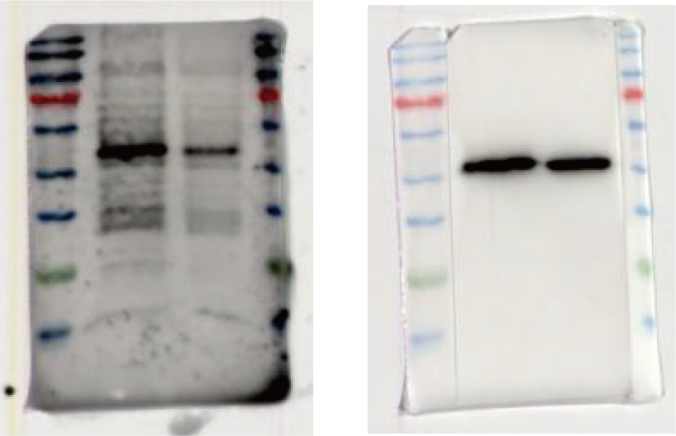


Western blot of p-Tau proteins in N2a/APP695 cells following ZB treatment, the molecular weight markers on the blot are 150 kDa, 135 kDa, 100 kDa, 75 kDa (red), 65 kDa, 45 kDa, 35 kDa, 25 kDa, 15 kDa, and 10 kDa from top to bottom.


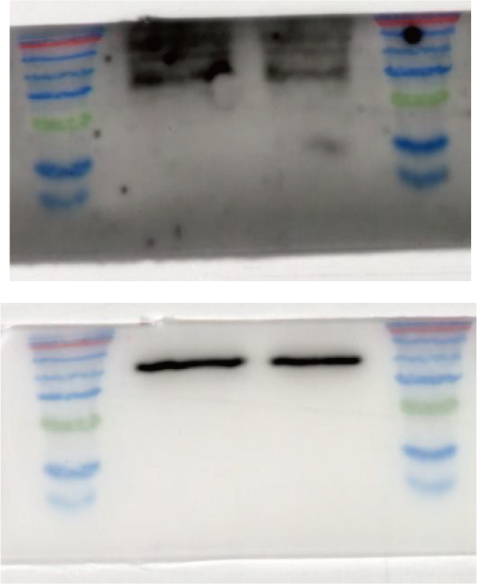


Western blot of Aβ proteins in N2a/APP695 cells following ZB treatment, the molecular weight markers on the blot are 150 kDa, 135 kDa, 100 kDa, 75 kDa (red), 65 kDa, 45 kDa, 35 kDa, 25 kDa, 15 kDa, and 10 kDa from top to bottom.


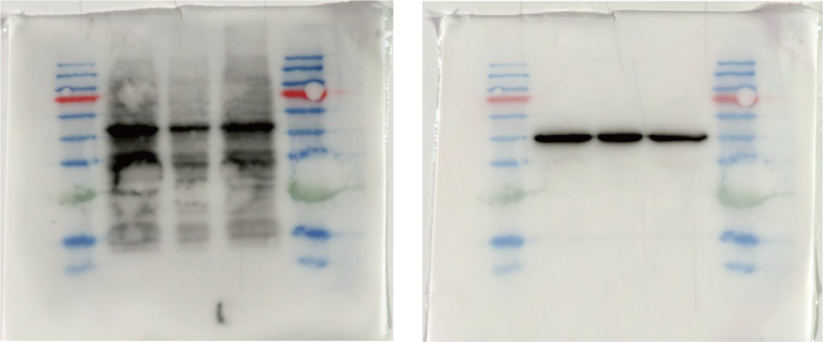


Western blot of p-Tau proteins in N2a/APP695 cells or RNAi cells following ZB treatment, the molecular weight markers on the blot are 150 kDa, 135 kDa, 100 kDa, 75 kDa (red), 65 kDa, 45 kDa, 35 kDa, 25 kDa, 15 kDa, and 10 kDa from top to bottom.


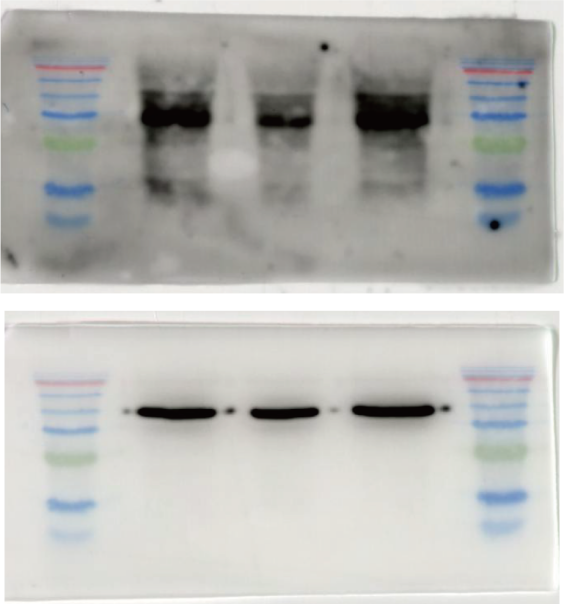


Western blot of Aβ proteins in N2a/APP695 cells or RNAi cells following ZB treatment, the molecular weight markers on the blot are 150 kDa, 135 kDa, 100 kDa, 75 kDa (red), 65 kDa, 45 kDa, 35 kDa, 25 kDa, 15 kDa, and 10 kDa from top to bottom.

**Supplementary_Material-S3**


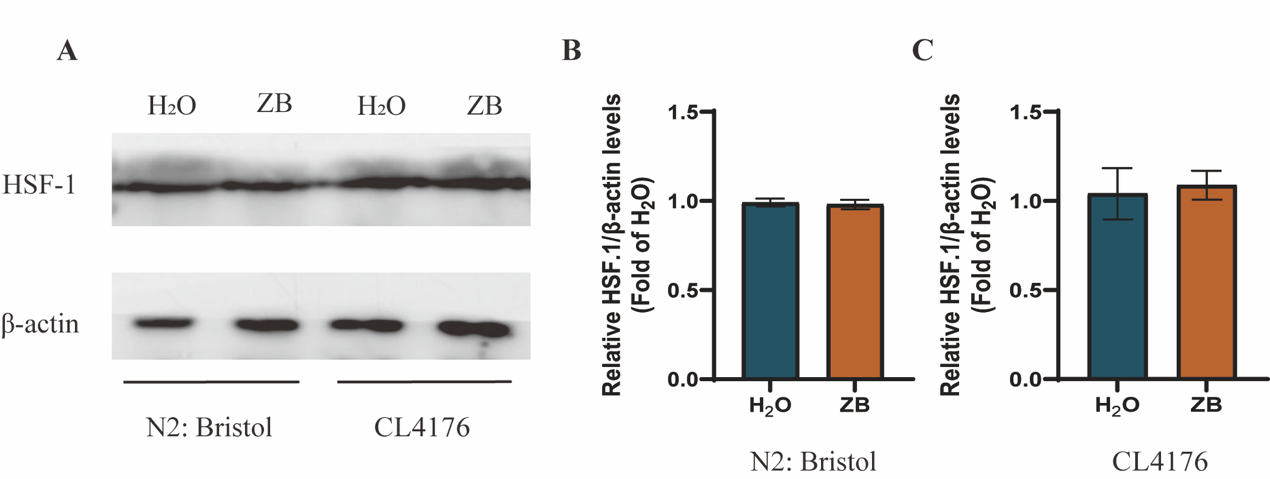


Western blot of HSF-1proteins in N2:Bristol and CL4176 *C. elegans* following ZB treatment.
